# Relationship between Glutamate Dysfunction and Symptoms and Cognitive Function in Psychosis

**DOI:** 10.3389/fpsyt.2013.00151

**Published:** 2013-11-26

**Authors:** Kate Merritt, Philip McGuire, Alice Egerton

**Affiliations:** ^1^Department of Psychosis Studies, Institute of Psychiatry, King’s College London, London, UK

**Keywords:** schizophrenia, psychosis, glutamate, NMDA, MRS, spectroscopy, imaging

## Abstract

The glutamate hypothesis of schizophrenia, proposed over two decades ago, originated following the observation that administration of drugs that block NMDA glutamate receptors, such as ketamine, could induce schizophrenia-like symptoms. Since then, this hypothesis has been extended to describe how glutamate abnormalities may disturb brain function and underpin psychotic symptoms and cognitive impairments. The glutamatergic system is now a major focus for the development of new compounds in schizophrenia. Relationships between regional brain glutamate function and symptom severity can be investigated using proton magnetic resonance spectroscopy (1H-MRS) to estimate levels of glutamatergic metabolites *in vivo*. Here we briefly review the 1H-MRS studies that have explored relationships between glutamatergic metabolites, symptoms, and cognitive function in clinical samples. While some of these studies suggest that more severe symptoms may be associated with elevated glutamatergic function in the anterior cingulate, studies in larger patient samples selected on the basis of symptom severity are required.

## Introduction

Accumulating evidence suggests that glutamatergic dysfunction may contribute to the pathogenesis of schizophrenia, and the symptoms and cognitive deficits associated with the disorder (1). The glutamatergic system presents an attractive therapeutic target, as dopaminergic antipsychotics have little effect on negative symptoms or cognitive impairment, yet these features are better predictors of social and functional outcome than positive symptoms (2, 3). Here we briefly review the existing evidence linking abnormal glutamatergic transmission to cognitive, negative, and positive symptoms of schizophrenia.

The observation that administration of antagonists at the *N*-methyl-d-aspartate glutamate receptor complex (NMDAR) such as phencyclidine (PCP) or ketamine to healthy volunteers induces effects which resemble aspects of schizophrenia symptomatology forms a cornerstone of the glutamate hypothesis of schizophrenia (4–6). NMDAR antagonists also worsen positive, negative, and cognitive symptoms in patients with schizophrenia (7, 8). While dopamine-stimulating drugs such as amphetamine also produce positive “psychotic”-like effects, negative-type symptoms, and cognitive deficits are far more prominently elicited by ketamine than amphetamine administration (9). Neuroimaging studies indicate that these effects of ketamine are mediated by changes in activity in the frontal and cingulate cortices and the thalamus (10–13).

While pharmacological studies have provided evidence linking NMDAR dysfunction to these symptom domains, direct associations between glutamatergic function and symptom severity may be provided by neuroimaging studies. In a single photon emission tomography study using the NMDAR radiotracer ^123^I-CNS-1261 in schizophrenia, the availability of NMDAR in the hippocampus was negatively associated with the severity of symptoms, especially negative symptoms (14). A ketamine challenge study in healthy volunteers using the same radiotracer linked regional NMDAR binding to the induction of negative (but not positive) symptoms, particularly in the thalamus (15). These approaches are currently limited by a lack of availability of suitable radiotracers (1).

An alternative is to use proton magnetic resonance spectroscopy (1H-MRS) to estimate the concentration of glutamatergic metabolites. MRI scanners with field strengths of 3 T or above can resolve glutamate, at least for the most part, from its metabolite glutamine (16). At lower field strengths glutamate and glutamine are reported in combination, as Glx. A limitation of 1H-MRS is that the glutamate concentration estimates are not specific to neuronal glutamate, and that changes in glutamate levels cannot be specifically attributed to altered neurotransmission over other metabolic processes (17). However the majority (∼80%) of glutamine synthesis reflects cycling of neurotransmitter glutamate (17), and clinical studies at 4 T in minimally treated patients with schizophrenia have reported higher Gln/Glu ratios (18) or higher glutamine levels (19) in the anterior cingulate cortex (ACC). This is consistent with increased glutamatergic neurotransmission, but could also result from a deficiency in the conversion of glutamine to glutamate. However the majority of 1H-MRS studies in psychosis have used field strengths <4 T and thereby were unable to accurately quantify glutamine concentrations. Thus, the results from these studies at lower field strengths cannot be specifically attributed to changes in glutamate neurotransmission. Nonetheless, increases in frontal glutamatergic neurotransmission in schizophrenia are broadly consistent with the NMDA receptor hypofunction hypothesis, as administration of NMDAR antagonists to rats increases glutamate release as detected by microdialysis (20), and 1H-MRS studies report elevated Gln/Glu ratios (21), or increased glutamate levels with no changes in glutamine (22). 1H-MRS studies with ketamine in man at 4 T reveal increased ACC glutamine (23), and at 3 T reveal increased ACC glutamate (24). One mechanism through which increases in glutamate release may occur is via NMDAR dysfunction on GABA-ergic interneurons, leading to the disinhibition of glutamatergic pyramidal cells, as described by the NMDAR hypofunction hypothesis (25).

Individual 1H-MRS glutamate studies in schizophrenia have produced some inconsistent findings. In general, studies at higher field strengths suggest that frontal glutamine function is elevated in the early stages of psychosis (18, 19), whereas the findings in chronic schizophrenia are more variable (26–30). This could reflect effects of antipsychotic medication. For example, one cross-sectional and one longitudinal study reported higher Glx levels in the PFC of unmedicated, but not medicated schizophrenia, in comparison to controls (31, 32), although longitudinal studies have reported no effect of medication on anterior cingulate glutamate or glutamine levels (18, 33, 34). It could be that symptom severity contributes to variability in glutamatergic metabolites between patients early or late in the illness, as samples involving patients in the early phase of psychosis usually comprise non- or minimally medicated patients who are relatively symptomatic, whereas studies in chronic schizophrenia often involve patients who have been treated for long periods and have less severe or more stable symptoms (1). In relation to this, a recent meta-analysis of 1H-MRS glutamatergic studies in major depressive disorder found lower levels of Glx in the ACC which were only significant when remitted patients were excluded from the analysis (35).

In order to better understand the possible relationships between 1H-MRS glutamate, glutamine, and Glx levels and symptom severity, we identified publications that have reported associations with severity along symptom domains. These included studies in individuals at risk of psychosis, and patients with first-episode psychosis or established schizophrenia (Table 1).

**Table 1:** **Summary of articles reporting high levels of glutamate metabolites associated with greater or lesser severity of symptoms**.

Reference	Field strength	Population	*n*	Brain region	Metabolite	Measure	Direction
Tandon et al. (54)	1.5	Familial high risk	23	Thalamus	Glx	SIPS	+
						CSS	NS
				Caudate	Glx	SIPS, CSS	+
				ACC	Glx	SIPS, CSS	NS
Yoo et al. (36)	1.5	GHR	22	ACC, DLPFC, thal	Glx	PANSS, BPRS	NS
de la Fuente-Sandoval et al. (37)	3	ARMS + drug naïve FE	36	Associative striatum, cerebellar cortex	Glu	PANSS, SIPS	NS
de la Fuente-Sandoval et al. (47)	3	FE	24	Associative striatum	Glu	PANSS	+
				Cerebellar cortex	Glu, Glx	PANSS	NS
Egerton et al. (38)	3	FE	32	ACC	Glu/Cr	PANSS negative	+
						PANSS positive	NS
				Thalamus	Glu/Cr	PANSS	NS
Ota et al. (48)	1.5	Chronic (+1 FE)	46	Inferior parietal	Glx	PANSS positive	+
				Middle frontal	Glx	PANSS	NS
Ohrmann et al. (42)	1.5	Chronic	43	ACC, DLPFC	Glx	PANSS, CDSS, CGI	NS
Tayoshi et al. (28)	3	Chronic	30	ACC, basal ganglia	Glu, Gln	PANSS	NS
Wood et al. (30)	3	Chronic	15	Dorsal, rostral cingulate	Glx	PANSS	NS
Kegeles et al. (31)	3	Chronic	32	mPFC (inclu ACC)	Glx	PANSS negative	NS
						PANSS positive	+ #
Szulc et al. (62)	1.5	Chronic	42	Frontal lobe	Glx/Cr	PANSS	+
Ohrmann et al. (41)	1.5	Chronic	39	DLPFC	Glx	PANSS	NS
Ongur et al. (50)	4	Chronic	17	ACC, POC	Gln/Glu	PANSS, MADRS, YMRS	NS
Stanley et al. (39)	1.5	FE 14wk treatment	37	DLPFC	Gln	SANS, SAPS	NS
Bartha et al. (40)	1.5	FE	10	mPFC (inclu ACC)	Glu, Gln	SANS, SAPS	NS
Theberge et al. (19)	4	FE	21	ACC, thal	Glu, Gln	SANS, SAPS	NS
Bustillo et al. (18)	4	FE Min treated	14	ACC, thal	Gln/Glu	SANS, SAPS	NS
Bustillo et al. (43)	4	Chronic	30	Whole brain slice	Glx	SANS	−
						SAPS	NS
Olbrich et al. (67)	2	FE	9	DLPFC	Glu	SANS, BPRS	−
				Hippocampus	Glu	SANS, BPRS	NS
Shirayama et al. (27)	3	Chronic	19	mPFC (inclu ACC)	Gln/Glu	SANS, BPRS	NS
Rowland et al. (44)	3	Chronic	20	mPFC, inferior parietal	Glx	SANS, BPRS	NS
Rowland et al. (46)	3	Chronic	21	ACC, CSO	Glx	SANS, BPRS	NS
Choe et al. (32)	1.5	Chronic	34	PFC	Glx/Cr	BPRS	+
Reid et al. (26)	3	Chronic	26	ACC	Glx	BPRS negative	−
						BPRS positive	NS
Reid et al. (45)	3	Chronic	35	Substantia nigra	Glx/Cr	BPRS	NS
Tandon et al. (54)	1.5	Familial high risk	23	ACC, thal, caudate	Glx	WCST	NS
Rusch et al. (53)	2	FE + Chronic	29	Hippocampus	Glu	WCST	+
Shirayama et al. (27)	3	Chronic	19	mPFC (inclu ACC)	Gln/Glu	WCST, DSDT	+
						Stroop, VF, IGT	NS
Ohrmann et al. (42)	1.5	Chronic	43	ACC	Glx	WCST	−
				ACC, DLPFC	Glx	AVLT	NS
Ohrmann et al. (55)	4	FE + Chronic	35	DLPFC	Glx	AVLT	−
Kegeles et al. (31)	3	Chronic	32	mPFC (inclu ACC)	Glx	N-back	NS
Reid et al. (26)	3	Chronic	26	ACC	Glx	RBANS	NS
Reid et al. (45)	3	Chronic	35	Substantia nigra	Glx/Cr	RBANS	NS
Rowland et al. (44)	3	Chronic	20	mPFC, inferior parietal	Glx	RBANS	NS
Rowland et al. (46)	3	Chronic	21	ACC, CSO	Glx	RBANS	NS
Bustillo et al. (43)	4	Chronic	30	Whole brain slice	Glx	Combined neuropsych	−
Yoo et al. (36)	1.5	GHR	22	ACC, DLPFC, thal	Glx	GAF	NS
Tibbo et al. (59)	3	GHR	20	mPFC	Glx/Cr	GAF	−
Egerton et al. (38)	3	FE	32	ACC	Glu/Cr	GAF	+
Egerton et al. (38)	3	FE	32	Thalamus	Glu/Cr	GAF	NS
Shirayama et al. (27)	3	Chronic	19	mPFC (inclu ACC)	Gln/Glu	GAF	NS
Tebartz van Elst et al. (60)	2	Chronic	21	DLPFC	Glu	GAS	+
Aoyama et al. (34)	4	Chronic	17	Thalamus	Total Gln + Glu	LSPR	−

*# Did not survive correction for the six comparisons (PANSS total, positive, and negative symptom subscales in two regions for each neurochemical)*.

*Index: + denotes a positive relationship where higher levels of glutamate were associated with greater symptom severity or worse overall functioning; − denotes a negative relationship where higher levels of glutamate were associated with lesser symptom severity or better overall functioning. NS, not significant; ACC, anterior cingulate cortex; POC, parieto-occipital cortex; DLPFC, dorsolateral prefrontal cortex; MPFC, medial prefrontal cortex; Thal, thalamus; CSO, centrum semiovale; GHR, genetic high risk; RBANS, repeatable battery for the assessment of neuropsychological status; WCST, Wisconsin card sorting test; DSDT, the digit span distraction test; TMT, trail making test; IGT, Iowa gambling task; VF, verbal fluency; AVLT, auditory verbal learning test; BPRS, brief psychiatric rating scale; GAF, global assessment of functioning; CGI, clinical global impression scale; GAS, global assessment scale; SIPS, structured interview for prodromal symptoms; CSS, Chapman schizotypy scales; CDSS, Calgary Depression Scale for Schizophrenia; LSPR, life skills profile rating*.

## Relationships between Glutamate Function and Positive Symptoms

Several studies have investigated associations between regional glutamatergic metabolite levels and the severity of positive psychotic symptoms (Table 1), and most found no association between regional Glx, glutamate or glutamine levels and positive symptom severity, in either high genetic or clinical risk populations (36, 37), first-episode psychosis (18, 19, 37–40) or chronic schizophrenia (26–28, 30, 41–46).

However, many of these studies involved small patient samples, relied on *post hoc* correlational analyses, and patients in whom the severity and/or the variance in severity of symptoms was low, due to either being sub-clinical threshold in the at risk studies or due to the presence of antipsychotic medication in established schizophrenia. A recent study pooling both medicated and unmedicated patients, where unmedicated patients possessed elevated Glx in medial prefrontal cortex (mPFC), detected an association between positive symptom severity and mPFC Glx, although this did not survive correction for multiple comparisons (31). Another study in the PFC found that treatment reduced the level of Glx/Cr in chronic patients, and associated the change in Glx with improvement in total BPRS score (32). Moreover a recent study found that 4 weeks of antipsychotic treatment in first-episode psychosis patients reduced Glx in the striatum, and this was associated with improvement in PANSS score (47).

A notable exception is the study of Ota et al. (48), which directly compared patients experiencing exacerbated psychotic symptoms to healthy controls and stable patients, and found that increases in Glx in inferior parietal white matter were specific to the group currently experiencing exacerbated psychotic symptoms (48). In line with this, our own studies which have compared glutamate levels in patients according to symptom severity have found higher ACC glutamate levels in first-episode psychosis patients who are still symptomatic following treatment compared to those in remission (38) and in patients with treatment-resistant schizophrenia compared to those who respond to medication (49). These differences may be independent of medication effects, as groups either did not differ according to medication (38, 49) or the symptomatic group were actually receiving higher medication doses (48), and as longitudinal studies have not reported changes in cortical glutamate in relation to changes in positive symptoms following antipsychotic treatment (18, 33, 34). This suggests that glutamate and Glx levels may be selectively elevated in patients whose positive symptoms are not well controlled by conventional antipsychotic medication.

## Relationships between Glutamate Function and Negative Symptoms

We recently reported that greater severity of PANSS negative symptoms was associated with higher levels of glutamate in the ACC in first-episode psychosis (38). Although there were several methodological differences, this contrasts with the study of Reid et al. (26), which reported that negative symptoms were associated with *lower* levels of ACC Glx in chronic schizophrenia. Other studies investigating correlations between ACC glutamatergic metabolites and negative symptoms have found no significant relationship (18, 19, 28, 42, 46, 50). We are not aware of any studies which have specifically compared regional levels of glutamatergic metabolites in patient groups selected according to differences in negative symptom severity.

As listed in Table 1, in brain regions other than the ACC many studies have failed to detect significant relationships between negative symptoms and regional glutamate, glutamine, or Glx levels in high genetic or clinical risk groups (36, 37), first-episode psychosis (18, 37, 39, 40), or chronic schizophrenia (27, 30, 31, 41, 44, 45, 48).

A general consideration of studies investigating relationships between glutamate markers and positive and negative symptoms is the scale used to score symptom severity. As detailed in Table 1, scores on a number of scales have been used and while these scales are highly correlated there are also some important differences in the clinical items included (51). As most studies examining relationships between brain glutamate measures and symptoms have relied on *post hoc* correlational analysis, it is of note that additional items are included on the SAPS/SANS compared to the PANSS, which in turn has additional items compared to the BPRS. Therefore use of the SAPS/SANS may be preferable as they provide a more detailed assessment of symptoms.

## Relationships between Glutamate Measures and Cognitive Dysfunction

Relatively few studies have investigated relationships between glutamate measures and cognitive dysfunction in schizophrenia. The most commonly investigated task has been the Wisconsin card sort test (WCST): in schizophrenia, deficits on this task are associated with abnormal activation in the ACC and DLPFC (52). In a study of 19 patients with chronic schizophrenia at 3 T, poor performance on the WCST was associated with higher Gln/Glu ratios in the mPFC (including the ACC) (27). In contrast, a larger study of 43 patients with chronic schizophrenia at 1.5 T found that ACC, but not DLPFC, Glx levels were positively associated with WCST learning potential (42). In first-episode psychosis, an association between hippocampal, but not DLPFC, glutamate, and WCST errors was reported (53), and in a small sample of 16 genetic high risk individuals, no correlations between WCST performance and Glx levels in the caudate, ACC, or thalamus were detected (54).

Other tasks investigated include the Stroop, digit span distractibility test, auditory verbal learning test (AVLT), N-back task, Iowa gambling task and verbal fluency test (Table 1). Of these, there are reports of a positive association between mPFC Gln/Glu ratio and impairments on the digit span distraction test, which probes short-term memory and selective attention (27), and of DLPFC Glx and verbal learning and memory on the AVLT (55). Overall, the findings have been inconsistent and further studies with larger sample sizes are required. While the above studies used a voxel of interest method, using whole brain slice proton echo planar spectroscopy at 4 T in 30 patients Bustillo et al. (43) detected a positive correlation between general cognitive performance in schizophrenia and Glx. Furthermore, subsequent path analyses suggested that the relationship between glutamate and cognitive performance may be associated with negative symptoms and unemployment (43).

The relationship between glutamate and cognition in schizophrenia has been further investigated by combining 1H-MRS with functional magnetic resonance imaging (fMRI) of the blood oxygen level dependent (BOLD) response to measure changes in regional brain activation as participants perform cognitive tasks. In subjects at clinical high risk of developing psychosis, reductions in thalamic glutamate were correlated with an elevated BOLD response in the prefrontal cortex during a verbal fluency task, whereas the converse association was observed in controls (56). In a study by Valli et al. (57), medial temporal lobe glutamate levels were correlated with hippocampal BOLD response during an episodic memory task in controls, whereas this coupling was absent in clinical high risk subjects (57). Finally one study showed that hippocampal Glx was related to inferior frontal gyrus activation during episodic memory in controls, and suggested that the absence of this positive coupling in medicated schizophrenia patients may underlie episodic memory deficits, reflecting the results seen in Valli et al. (57, 58). Further investigation of the relationships between regional levels of glutamatergic metabolites and abnormalities in regional brain activation during cognitive tasks may provide a more sensitive means to characterize the relationship between glutamate and cognition in schizophrenia.

## Relationships between Glutamate Measures and Social and Occupational Functioning

Several studies have also reported correlations between brain glutamate levels and overall level of social and occupational functioning (Table 1). In genetically high risk subjects, lower levels of Glx/Cr in the mPFC were associated with lower levels of overall functioning (59). A longitudinal study at 4 T showed that loss of glutamate and glutamine in the thalamus, but not the ACC, over 7 years since first presentation correlated with impaired social functioning (34). In contrast, in first-episode psychosis higher levels of glutamate in the ACC, but not the thalamus, were associated with worse overall functioning (38), and in chronic schizophrenia higher DLPFC glutamate levels were also associated with worse overall functioning (60). Other studies have found no association between glutamate measures and overall functioning in chronic schizophrenia (27) or genetic high risk subjects (36).

## Summary and Future Directions

1H-MRS studies relating regional glutamate measures to symptoms in schizophrenia have produced inconsistent findings. There may be several methodological reasons for this, including differences in the brain region investigated, and between samples such as medication, illness stage, and symptom severity. The use of sub-optimal technical approaches such as low field strengths together with small sample sizes may also underlie the conflicting findings. A recent study at 4 T indicates that elevations in the ratio of Gln/Glu are present in schizophrenia patients, consistent with elevated glutamatergic neurotransmission (18). However the majority of 1H-MRS studies in schizophrenia use field strengths <4 T which cannot reliably measure glutamine, and glutamate and Glx measures cannot be specifically attributed to glutamate neurotransmission (17). The increasing availability of higher field strength scanners may resolve some of the apparent inconsistencies in the literature.

The majority of studies that have investigated relationships between glutamate levels and symptom severity have applied correlational analysis, usually *post hoc* to the main study findings. A few studies have directly compared glutamatergic metabolite levels in patient groups on the basis of symptom severity, specifically those who were or were not currently experiencing symptom exacerbation (48), were or were not in symptomatic remission following initial treatment (38) or did or did not have treatment-resistant illness (49). All found higher levels of glutamate or Glx in the more symptomatic patient group.

The field would benefit from further studies pre-selecting groups of patients who differ in severity of negative symptoms or cognitive impairment, or longitudinal studies comparing within-subjects glutamate levels during periods of illness stability compared to relapse. Studies of the relationships between cognitive dysfunction and glutamate in schizophrenia may benefit from combination with fMRI to determine the efficiency of glutamate in supporting networks that subserve cognitive function. To date there are only a handful of published studies of this type. Finally, relationships between glutamate levels and symptom severity may be indirect; for example Stone et al. showed that hippocampal glutamate may interact with striatal dopamine to determine risk of psychosis (61), and a path analysis has suggested that negative symptoms may be secondary to poor cognition associated with low brain Glx (43).

One question is whether baseline levels of glutamate, glutamine, or Glx are predictive of subsequent outcome or response to treatment in schizophrenia. A recent study showed that elevated frontal Glx at baseline was associated with poor response after 4 weeks of antipsychotic treatment (62). This is consistent with the above findings of glutamatergic elevations in patients with schizophrenia whose symptoms have not responded well to treatment (38, 49).

The suggestion that symptoms that do not respond well to conventional antipsychotic treatment may have a glutamatergic basis (38, 49) warrants further investigation, as compounds that target the glutamatergic system may have particular efficacy in these patients. Meta-analyses conclude that of the agents which may improve NMDA receptor-mediated neurotransmission, the NMDAR co-agonist d-serine and the glycine transporter type 1 inhibitor sarcosine reduce total and negative symptoms as an adjuvant to antipsychotic medication (63, 64). Lamotrigine, which may inhibit glutamate release, blocks the psychomimetic effects of ketamine in healthy volunteers (65) and small trials of lamotrigine in chronic, often treatment-resistant or clozapine-treated patients found that it is beneficial in reducing symptoms (63, 66). Further work is required to determine the relationship between regional glutamate concentrations and the expression of symptoms at different stages of psychotic illness. This area may benefit from meta-analyses of the previously published findings and from new studies selecting patient samples *a priori* on the basis of symptom severity.

## Conflict of Interest Statement

The authors declare that the research was conducted in the absence of any commercial or financial relationships that could be construed as a potential conflict of interest.
